# Identification and Characterization of Argonaute Protein, Ago2 and Its Associated Small RNAs in *Schistosoma japonicum*

**DOI:** 10.1371/journal.pntd.0001745

**Published:** 2012-07-31

**Authors:** Pengfei Cai, Xianyu Piao, Nan Hou, Shuai Liu, Heng Wang, Qijun Chen

**Affiliations:** 1 MOH Key Laboratory of Systems Biology of Pathogens, Institute of Pathogen Biology, Chinese Academy of Medical Sciences and Peking Union Medical College, Beijing, People's Republic of China; 2 Department of Microbiology and Parasitology, Institute of Basic Medical Sciences, Chinese Academy of Medical Sciences and School of Basic Medicine, Peking Union Medical College, Beijing, People's Republic of China; 3 Key Laboratory of Zoonosis, Ministry of Education, Institute of Zoonosis, Jilin University, Changchun, People's Republic of China; Queen's University Belfast, United Kingdom

## Abstract

**Background:**

The complex life cycle of the genus *Schistosom*a drives the parasites to employ subtle developmentally dependent gene regulatory machineries. Small non-coding RNAs (sncRNAs) are essential gene regulatory factors that, through their impact on mRNA and genome stability, control stage-specific gene expression. Abundant sncRNAs have been identified in this genus. However, their functionally associated partners, Argonaute family proteins, which are the key components of the RNA-induced silencing complex (RISC), have not yet been fully explored.

**Methodology/Principal Findings:**

Two monoclonal antibodies (mAbs) specific to *Schistosoma japonicum* Argonaute protein Ago2 (SjAgo2), but not SjAgo1 and SjAgo3, were generated. Soluble adult worm antigen preparation (SWAP) was subjected to immunoprecipitation with the mAbs and the captured SjAgo2 protein was subsequently confirmed by Western blot and mass spectrometry (MS) analysis. The **s**mall RNA population associated with native SjAgo2 in adult parasites was extracted from the immunoprecipitated complex and subjected to library construction. High-through-put sequencing of these libraries yielded a total of ≈50 million high-quality reads. Classification of these small RNAs showed that endogenous siRNAs (endo-siRNAs) generated from transposable elements (TEs), especially from the subclasses of LINE and LTR, were prominent. Further bioinformatics analysis revealed that siRNAs derived from ten types of well-defined retrotransposons were dramatically enriched in the SjAgo2-specific libraries compared to small RNA libraries constructed with total small RNAs from separated adult worms. These results suggest that a key function of SjAgo2 is to maintain genome stability through suppressing the activities of retrotransposons.

**Conclusions/Significance:**

In this study, we identified and characterized one of the three *S. japonicum* Argonautes, SjAgo2, and its associated small RNAs were found to be predominantly derived from particular classes of retrotransposons. Thus, a major function of SjAgo2 appears to associate with the maintenance of genome stability via suppression of retroelements. The data advance our understanding of the gene regulatory mechanisms in the blood fluke.

## Author Summary

Schistosomiasis, a chronic disease caused by agents of the genus *Schistosoma*, still afflicts more than 230 million people worldwide. The genomes of the three major pathogenic blood flukes, *Schistosoma japonicum*, *Schistosoma mansoni*, and *Schistosoma haematobium*, have been decoded as valuable entities for a systematic dissection of the biological characteristics of the parasites. Transposable elements constitute a major component in the genome of schistosomes and have been recognized as remnants of evolutionary events, but some of them are still active today. Thereby, the activity of these active mobile genetic elements should be restricted by elaborate mechanisms to protect genome stability. Our study showed that one of the three *S. japonicum* Argonaute proteins, SjAgo2, is involved in such mechanisms. By using specific mAb, native SjAgo2 protein was immunoisolated from a soluble adult worm antigen preparation, and its associated small RNAs were extracted for deep sequencing. We found that SjAgo2 is mainly associated with particular types of retrotransposon-derived siRNAs. For instance, siRNAs generated from 10 classes of well-defined retrotransposons were significantly enriched in the SjAgo2-specific libraries. Thus, a major function of Ago2 in *S. japonicum* is proposed to be the maintenance of genome stability via retrotransposon suppression. Our findings advance understanding of the putative gene regulatory mechanisms in a flatworm parasite.

## Introduction

Schistosomiasis is a chronic debilitating disease caused by the parasitic blood flukes of the genus *Schistosoma*, which afflicts more than 230 million individuals in 77 endemic countries (http://www.who.int/mediacentre/factsheets/fs115/en/index.html). The schistosomes have a complex developmental life cycle characterized by an asexual multiplication phase (mother sporocysts and daughter sporocysts) in the molluscan hosts and a sexual development and reproduction phase (lung-stage schistosomula, juvenile, adult male and female worms, and eggs) in mammalian hosts, as well as the aquatic free-swimming phase including miracidia and cercariae [1]. It is well known that the schistosome parasites undergo dramatic morphological transformation and rapid physiological adaptation to its life niche during development [2], which is essentially controlled by subtle gene regulatory mechanisms [1], [3]–[6]. The decoding of the genomes of the three major pathogenic blood flukes, S*chistosoma japonicum*, *Schistosoma mansoni*, and *Schistosoma haematobium*, has provided a valuable entity for a systematic dissection of the parasite biology [4], [7], [8].

In the past decade, small non-coding RNAs (sncRNAs) have emerged as critical regulators of gene expression both at transcriptional and post-transcriptional levels in metazoans, plants, fungi, and viruses [9]–[12]. In schistosomes, sncRNA repertoires at different developmental stages of the parasites have been revealed [13]–[19]. Both microRNAs (miRNAs) and small endogenous interfering RNAs (endo-siRNAs) are expressed in a stage- and gender-biased manner. MiRNA transcripts are generated primarily from the intergenic regions of the genome, whereas endo-siRNAs are principally originated from the transposable elements, including transposons and retrotransposons. The preferential expression of these sncRNAs in different developmental stages and sexes suggests that they play distinct roles in modulating development, maturation, and reproduction of the parasite [14]–[16], [18], [19].

To exert their activities, sncRNAs must be selectively loaded onto their relevant machinery, the RNA-induced silencing complex (RISC), and guide the RISC to their complementary templates. Argonaute family proteins are at the heart of RISCs, which can be divided into Ago and PIWI subfamilies [20], and a third clade, termed ‘group III Argonautes’ is worm-specific for binding secondary siRNAs [21]. Although small RNA pathways are evolutionally conserved, the number of Argonaute genes varies dramatically in different organisms, ranging from one in the fission yeast *Schizosaccharomyces pombe* to twenty-seven in the nematode *Caenorhabditis elegans* [21]. Different small RNA regulatory pathways (SRRPs) may be mediated by one Argonaute protein, such as metazoan-like Argonaute in the single-cell parasite *Toxoplasma gondii* [22], or entangled with multiple Argonaute proteins, which compete and collaborate with each other to form regulatory networks [23]–[25]. In *S. mansoni*, four Argonaute proteins were identified by two groups mainly based on bioinformatic analysis, but SmAgo3 and SmAgo4 seemed to be generated from an alternatively spliced mRNA [26]. Argonaute orthologs in *S. japonicum* (SjAgos) have been also reported by two groups [27], [28]. Both of them tried to determine the full-length sequences of the three Argonaute proteins and described the molecular characteristics of SjAgos. Chen *et al*. also reported the differential expression of *SjAgos* during the parasite development and suggested that SjAgos coordinated in different SRRPs may be involved in regulating schistosome development [27]. In addition, no PIWI homologue was identified in *S. japonicum*, though it was found in its closely related genus *Schmidtea mediterranea* [29], [30].

Although abundant small non-coding RNAs have been identified in schistosomes, the authentic function of Argonaute proteins in different SRRPs is still largely unknown. SjAgo1 has been previously speculated to participate in the miRNA pathway due to its high homology with miRNA-associated Argonautes in flies, humans, and worms, although experimental support for this idea is still lacking [31]. In this study, by using SjAgo2-specific mAb (27A9), native SjAgo2 complex and the associated small RNAs in the parasite were identified and deeply analyzed. Classification of the small RNAs led us to propose that suppression of parasitic retrotransposons within the genome may be the primordial biological function of SjAgo2.

## Materials and Methods

### Parasites and animals

The parasite-infected *Oncomelania hupensis* were provided by Jiangxi Institute of Parasitic Diseases, Nanchang, China. The freshly released cercariae of *S. japonicum* were harvested for Total RNA isolation. To obtain hepatic schistosomula and adult worms, New Zealand White rabbits were percutaneously infected with *S. japonicum* cercariae (1000 to 1500 per rabbit). Hepatic schistosomula were isolated from the rabbits at 2 weeks post-infection, while mixed adult worms were obtained after 6-weeks post infection by hepatic-portal perfusion. Male and female adult worms were manually separated with the aid of a light microscope. Eggs were isolated from liver tissues of infected rabbits by enzyme digestion method [32]. All procedures performed on animals within this study were conducted following animal husbandry guidelines of the Chinese Academy of Medical Sciences and with permission from the Experimental Animal Committee of Chinese Academy of Medical Sciences with the Ethical Clearance Number IPB-2011-6.

### Total RNA isolation and quality control

Total RNAs of *S. japonicum* at different developmental stages (cercariae, hepatic schistosomula, separated adult male and female worms, and eggs) were extracted using RNeasy Mini kit (QIAGEN) and the contaminating genomic DNA was removed from RNA samples with TURBO DNA-free™ kit (Ambion, CA, USA). RNA quantification and quality control was conducted by denaturing agarose gel electrophoresis and Nanodrop ND-1000 spectrophotometer (Nanodrop Technologies, Wilmington, DE).

### 5′ RACE

One µg total RNA from *S. japonicum* adult worms was used to synthesize the first strand cDNA using SuperScript™ III Reverse Transcriptase Kit (Invitrogen, CA, USA), with oligo (dT) 15 primer. The 5′ UTR of *SjAgo2* gene was amplified with a SMART RACE cDNA Amplification Kit according to the manufacturer's instructions (Clontech, CA, USA). The amplicons were cloned into T-Vector and sequenced. The primers used for 5′ RACE were listed in Table S1.

### Quantitative RT-PCR

xQRT-PCR was performed to quantitate the expression level of *SjAgo1*, *SjAgo2*, and *SjAog3* transcripts at different developmental stages of the parasite and between separated adult worms. For each sample, 1 µg total RNA was reverse transcribed into first-strand cDNA using SuperScript™ III Reverse Transcriptase Kit (Invitrogen) with Oligo dT (15) primer by incubation for 5 min at 25°C, 60 min at 50°C, and 15 min at 70°C. The resulting cDNA products were diluted 20-fold with nuclease-free water before qPCR. Each 25 µl PCR reaction contained 12.5 µl of 2×Brilliant II SYBR Green QPCR Master Mix (Agilent, USA), 1 µl cDNA, 1 µl of the forward and reverse primer pair (Table S1), and 10.5 µl of sterile water. The PCR conditions included 40 cycles with denaturation at 95°C for 30 s, followed by annealing and extension at 60°C for 1 min. A dissociation step (95°C for 15 s, 60°C for 1 min, 95°C for 15 s, and 60°C for 15 s) was added to confirm the amplification specificity for each gene. The PCR products were separated on a 2.5% agarose gel to confirm the presence of a single band with the expected size. Quantification of the expression for each *SjAgo* gene during the parasite development was performed by normalizing against a novel house-keeping gene, *PSMD4* (26S proteasome non-ATPase regulatory subunit 4, GenBank accession number: FN320595) [33] and applying the comparative 2^−ΔΔCt^ method using the software SDS 1.4.

### Preparation of SWAP for immunoprecipitation

The SWAP (soluble adult worm antigen preparation) was prepared mainly as previously described with minor modification. Briefly, the *S. japonicum* adult worms were washed in PBS for five times to reduce contamination of host components, homogenized on ice in lysis buffer containing 20 mM Tris-HCl (pH 7.4), 200 mM NaCl, 2.5 mM MgCl_2_, 0.05% NP-40, EDTA-free protease inhibitor cocktail (Roche) and RNasin (Promega) at a final concentration of 0.1 U/µl. The homogenate was then centrifuged at 14,000 g for 10 min at 4°C and the supernatant was collected carefully to avoid the top lipid layer. This procedure was repeated until the supernatant was clear. The supernatant was stored at −80°C for further use.

### Construction of the plasmids for generation of recombinant SjAgos

The DNA fragments encoding tSjAgo1 (aa198-1009), SjAgo2 (aa1-935), and SjAgo3 (aa1-923) were amplified from *S. japonicum* adult worm cDNA using high fidelity Phusion DNA polymerase (Finnzymes Oy, Finland) with *Kpn*I and *Not*I endonucleases site added at their 5′ and 3′ terminus, respectively (Primer sets were listed in Table S1). The PCR was performed with an initial denaturation for 1 min at 98°C. Ten PCR cycles were performed as follows: 98°C for 8 s, 50°C for 30 s and 72°C for 1 min, followed by another twenty PCR cycles: 98°C for 8 s, 55°C for 30 s and 72°C for 1 min, with a final extension at 72°C for 5 min. The amplicons were digested with *Kpn*I and *Not*I restriction endonucleases, and cloned into pcDNA3-FLAG3C vector. The recombinant plasmids were transformed into DH5α (DE3) *Escherichia coli* and positive clones were selected for sequencing. The correct recombinant plasmids were designated as FLAG-tSjAgo1, FLAG-SjAgo2, and FLAG-SjAgo3, respectively.

### Cell culture and transfection

To generate Flag-tagged recombinant SjAgos, human 293T cells were grown in Dulbecco's modified Eagle's medium supplemented with 2 mM L-glutamine and 10% fetal bovine serum. 293T cells were transfected at 90% confluency in 60-mm dishes with 8 µg of FLAG-tSjAgo1, FLAG-SjAgo2, FLAG-SjAgo3, or the empty vector, respectively, using Lipofectamine™ 2000 (Invitrogen). The cells were further cultured for 36 h at 37°C in a 5% CO_2_ incubator. Cell extracts were prepared in 200 µl lysis buffer containing 20 mM Tris-HCl (pH 7.5), 150 mM NaCl, 1% Triton X-100, and protease inhibitors.

### Production of mouse monoclonal antibodies to SjAgo2

Monoclonal antibodies 27A9 and 11E8 to SjAgo2 protein were produced by Abmart Inc (Shanghai, China). Briefly, the optimal peptide immunogens were selected from SjAgo2 by an in-house peptide selection database called Antibody Designer. Two cDNA fragments encoding aa1-232 and aa34–305 of SjAgo2 were subcloned into pET30a vector (Novagen) between *BamH*I and *Hind*III endonuclease sites. The recombinant plasmids were transformed into BL21 (DE3) *E. coli*. Two His-tag recombinant proteins were then expressed in *E. coli* and purified with Ni-NTA agarose beads. Six BALB/c mice were immunized subcutaneously with the peptides. Spleen cells obtained from immunized mice were fused with SP2/0 myeloma cells according to the standard procedure. Positive hybridomas were cloned, and immunoglobulin G (IgG) was purified by protein G affinity chromatography from ascite liquid.

### Immunoprecipitation and Western blot analysis

Immunoprecipitations were carried out essentially as described by Kiriakidou *et al*. [34]. For immunoprecipitation of endogenous SjAgo2 protein, a procedure of sequential depletion by absorption was adapted. One ml SWAP was first mixed with 100 µl of Protein-A/G agarose slurry (50%) (Abmart, Shanghai, China) and incubated at 4°C for 2 h with gentle rotation (Mock). After centrifugation at 2,500 rpm for 5 min, the supernatant was recovered and subsequently mixed with 4 µg normal mouse IgG (Santa Cruz Biotechnology) and incubated with gentle rotation at 4°C for 2 h. Then, 40 µl of Protein-A/G agarose was added and continually incubated at 4°C for another 2 h. The agarose beads were collected by centrifugation for 5 min at 2,500 rpm (moIgG IP). The supernatant was divided into equal parts and respectively mixed with 4 µg of 11E8 or 27A9 mAb, and gently incubated at 4°C for 4 h. The immunocomplex were captured by addition of 20 µl of Protein-A/G agarose beads and gently rotating for 2 h at 4°C. The beads were collected by centrifugation for 5 min at 2,500 rpm (mAb IP). The beads in the three sequential IP assays (without antibodies, with moIgG, and with specific mAbs) were further washed with 1 ml ice-cold lysis buffer for 5 times and resuspended with 1×SDS loading buffer. The protein samples were boiled for 10 min. After centrifugation, the supernatant was collected and used for further analysis.

Western blot analysis was performed as previously described [35]. Cell extracts with over-expressed tSjAgo1, SjAgo2, SjAgo3, SWAP, as well as immunoprecipitates were separately mixed with SDS-PAGE loading buffer and separated on SDS-PAGE gels, and transferred to the PVDF membrane. The membrane was blocked with 5% SMP in TBS for 90 min at room temperature. Anti-Flag mAb M2 (1∶2,000 dilution, Sigma) or anti-SjAgo2 mAbs (at a final concentration of 10 µg/ml) was used for detection of the target proteins. The HRP (horseradish peroxidase)-conjugated goat anti-mouse IgG (Zhongshan, China) at a dilution of 1∶10,000 was used as a secondary antibody and signal was detected with a luminol-based chemiluminescent substrate (CSN).

### Orbitrap Mass Spectrometry Analysis

To confirm that SjAgo2 was truly precipitated by the mAbs, two IP and MS assays were performed. In the first assay, the immunocomplex directly precipitated by mAb 27A9 from SWAP was resolved on a 10% SDS-PAGE gel and visualized by Coomassie Brilliant Blue staining. Protein bands with different molecular weights (>170 kDa, 130–170 kDa, 90–130 kDa, 70–80 kDa, 60–70 kDa, and 42–52 kDa) were excised and subjected to Orbitrap MS analysis. In the second assay, SWAP was sequentially incubated with Protein-A/G agarose beads (Mock), normal mouse IgG, and eventually with mAb 27A9. The immunoprecipitates were resolved on a 10% SDS-PAGE gel. Protein bands with sizes of ≈70–90 kDa and ≈90–120 kDa were excised from the gel (Figure S1) and digested with trypsin. The resulting peptides were analyzed by Orbitrap MS and identified by blasting against the protein datasets of *S. japonicum* downloaded from SDSPB (http://lifecenter.sgst.cn/schistosoma/en/schdownload.do) and Uniprot (http://www.uniprot.org/uniprot/?query=taxonomy%3a6182&format=).

### Small RNA library construction and sequencing

The SjAgo2 associated small RNAs were extracted as previously described [34]. RNA quantification and quality were evaluated by an Agilent 2100 Bioanalyzer (Figure S2). Small RNA libraries were constructed mainly as previously described [18]. Briefly, RNAs between 15–40 nucleotides (nt) were excised from a 15% TBE urea polyacrylamide gel electrophoresis (PAGE). The RNA sample was purified and their 5′ and 3′ termini were ligated with Illumina's proprietary adapters, which was further used as templates to synthesize first-strand cDNA. The cDNA was amplified by PCR with a high fidelity Phusion DNA polymerase and the Illumina's small RNA primer set. The libraries were sequenced on the Illumina Genome Analyzer II platform at the BGI (Beijing Genomics Institute, Shenzhen, China). IP assays were performed from two independent biological repeats with mAb 27A9, and the RNAs were separately applied for library construction and sequencing. The two libraries were designated as SP1 and SP2, respectively.

### Mapping sequence reads to the reference genome

Raw datasets produced by Solexa sequencing from the two libraries were tagged and pooled. Clean reads were obtained after removing of the low quality reads, adaptor null reads, insert null reads, 5′ adaptor contaminants, and reads with ployA tail. Adapter sequences were trimmed from 5′ and 3′ ends of clean reads. All identical sequences were counted and merged as unique sequences. These unique reads affiliated with read counts were mapped to the *S. japonicum* genome draft (sjr2_contig.fasta) (http://lifecenter.sgst.cn/schistosoma/en/schdownload.do) using the program SOAP version 2.20 [36].

### Bioinformatic analysis of small RNA libraries

First, we investigated the length distribution of small RNA reads in the two libraries that perfectly matched the genome draft of *S. japonicum*, and the small RNAs were categorized by the bioinformatic pipeline as described [18]. Afterwards, an alternative bioinformatic pipeline was designed to classify the small RNA reads that perfectly matched the reference genome. Briefly, the reads were matched to the transposable elements in the *S. japonicum* genome predicted by using REPET software (http://urgi.versailles.inra.fr/index.php/urgi/Tools/REPET), in an order of LINE (Long Interspersed Elements), SINE (Short Interspersed Elements), LTR (Transposable elements with Long Terminal Repeats), TIR (Terminal inverted repeat), MITE (Miniature inverted-repeat transposable elements), and unknown TE. The remaining small RNAs were aligned to *S. japonicum* predicted mRNA sequences (sjr_mRNA.fasta) downloaded from SDSPB using SOAP 2.20 aligner, and perfectly matched reads were retained as mRNA related siRNA. Next, the endo-siRNAs depleted reads were then BLAST-searched against the 78 known mature miRNAs of *S. japonicum* deposited in Sanger miRBase [37], [38] (Release 17) using the program Patscan [39], and further BLAST-searched against the conserved and novel *S. japonicum* miRNAs reported in our previous study [18]. Finally, homologs to rRNA, tRNA, snoRNA, and other small RNAs [40] were filtered and the remaining reads were labeled as unknown small RNAs.

To further characterize the small RNAs identified, full length sequences of 29 classes of retrotransposons [4], [41]–[43] were retrieved from the NCBI GenBank database [44]. The small RNA reads from the SP1, SP2, SjM, and SjF libraries were mapped to these retrotransposons. The abundance of these retrotransposon-derived siRNAs was reflected based on their expression values (TPM, transcripts per million). A set of graphs depicting the distribution and abundance of retrotransposon-derived siRNAs were further constructed as previously described [18]. Briefly, the expression of each base on these TEs was the sum of the TPM value of siRNAs that mapped to the position. A proper bin (10 or 50 bases) was then selected based on the length of TE sequences, and the average expression value was calculated for each bin.

## Results and Discussion

### Genes encoding the three Argonaute paralogues are differentially expressed in *S. japonicum*

**Figure 1:**
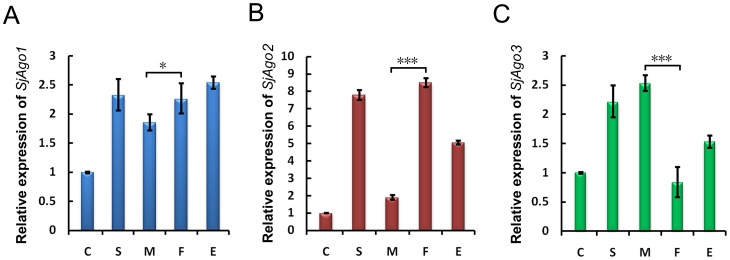
Trnascriptional analysis of three *S. japonicum* Argonaute genes. Relative levels of *SjAgo1* (**A**), *SjAgo2* (**B**), and *SjAgo3* (**C**) transcripts at different life stages (C, cercariae; S, hepatic schistosomula; M, male adult worms; F, female adult worms; E, eggs) were detected by qRT-PCR performed with three technical replicates. Transcriptional levels were calibrated based on the comparative 2^−ΔΔCt^ method using the housekeeping gene *SjPSMD4* as an endogenous control, and normalized to the expression in the cercarial stage. Error bars represent the standard deviations of the mean from the three technical replicates. The Student's *t*-test was employed to analyze the differential expression of *SjAgos* between male and female adult worms. *, *p*<0.05; ***, *p*<0.001. Here only the significance in difference between male and female parasite is indicated.

To investigate the possibility of functional or stage specificity of the three Argonaute paralogues in *S. japonicum*, we determined the transcription levels of the three Argonaute genes in the parasite before and after host invasion using qRT-PCR with that of 26S proteasome non-ATPase regulatory subunit 4 (*PSMD4*) as an endogenous control [33]. The overall expression level of the three genes was much lower in cercariae than in other stages within the host (Figure 1). This observation suggests that the SjAgos were mainly functional in the late developmental stages of the parasite. The expression of *SjAgo1* in eggs, miracidium, cercariae, schistosomula, and adult worms has been reported earlier by Lou *et al*. [28]. Even though the trend of the expression of *SjAgo1* was found similar between the two studies, our results were more profound than those reported previously. The difference was most likely caused by the different endogenous controls used in the two studies. The *SjGAPDH* gene was recently found to be unstably transcribed during the parasite development, which means that it is not suitable as an endogenous transcriptional control [33]. *SjAgo2* and *SjAgo3* presented a reversed expression pattern between male and female adult worms (Figure 1B and C). The expression of *SjAgo2* was up-regulated in schistosomula and female parasites, whereas *SjAgo3* was highly expressed in schistosomula and male worms. Previously, Chen *et al.* reported that the expression of *SjAgo1* was significantly higher in eggs than that in adult worms and the expression of *SjAgo2* and *SjAgo3* was not significantly different between male and female parasites [27]. This inconsistency is likely due to different experimental conditions, especially the endogenous controls applied in the two studies. However, it cannot be ruled out that the parasite strains in the two studies might be different.

### Determination of the full-length of SjAgo2

The two groups mentioned above also reported their analysis on the Argonaute family members in *S. japonicum* but with different results [27], [28]. In light of the uncertainty of the size of SjAgo2 protein, we performed 5′ RACE to determine the N-terminal region of the protein, for the C-terminus of the protein has been definitively defined. We confirmed that the full-length SjAgo2 protein contains 935 amino acids as reported by Chen *et al*. [27], but not 945 amino acids as reported by Luo *et al*. [28].

### Generation of specific mAbs to SjAgo2

**Figure 2:**
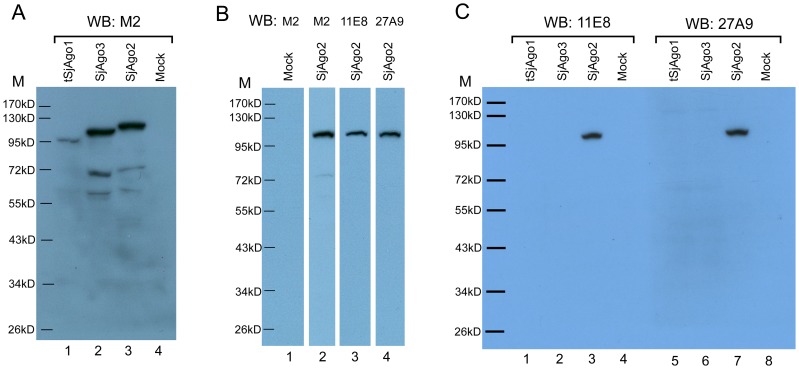
The expression of SjAgo proteins in eukaryotic cells and specific recognition by mAbs 11E8 and 27A9 raised against SjAgo2. **A.** The expression of Flag-tagged *S. japonicum* Argonaute proteins in 293T cells was detected by Western blot analysis using anti-Flag mAb M2. Lane 1, tSjAgo1 (aa198-1009); lane 2, SjAgo3; lane 3, SjAgo2; lane 4, Mock: Cells transfected with the empty vector. **B.** Over-expression of SjAgo2 was recognized by mAbs. Lane 1, Mock; lane 2–4, SjAgo2 was recognized by anti-Flag mAb M2, 11E8, and 27A9, respectively. **C.** Differential recognition of over-expressed SjAgos by mAb 11E8 or 27A9. Left panel, over-expressed SjAgos detected by Western blot with mAb 11E8. Right panel, over-expressed SjAgos detected by Western blot with mAb 27A9. M, prestained protein ladder SM0671.

In order to obtain specific mAbs against SjAgo2, two optimal peptide immunogens, aa1-232 and aa34-305 of SjAgo2, that avoided the major homologous regions with SjAgo3, were selected for immunizing BALB/c mice and two mAbs, 11E8 and 27A9, were generated. To determine the specificity of the mAbs against SjAgo2, we cloned the ORFs of the three Argonaute genes in the eukaryotic vector pcDNA3-FLAG3C, and the SjAgos were expressed in human 293T cells. Western blot analysis confirmed that SjAgo2 and SjAgo3, but not SjAgo1, were expressed in the 293T cells. Next, we tried to express a truncated form of SjAgo1 (tSjAgo1, aa198-1009), since the N-terminus of *SjAgo1* displayed very low similarity with SjAgo2 and SjAgo3 [27], it is unlikely that the mAb to the N-terminus of SjAgo1 would cross react with SjAgo2 and SjAgo3. The tSjAgo1 was successfully expressed in 293T cells, though the expression level was relatively lower than that of SjAgo2 and SjAgo3 (Figure 2A). The recognition of the recombinant SjAgo2 by mAb 11E8 or 27A9 was confirmed by Western blot analysis (Figure 2B). To determine whether both mAbs would cross-react with SjAgo1 and SjAgo3, equal amounts of the recombinant tSjAgo1, SjAgo2, and SjAgo3 were loaded in each lane (Figure S3). The blot was further detected by mAb 11E8 or 27A9, and both mAbs only specifically recognized SjAgo2, but not tSjAgo1 and SjAgo3 (Figure 2C).

### Identification of native SjAgo2 in SWAP immunoprecipitates

**Figure 3:**
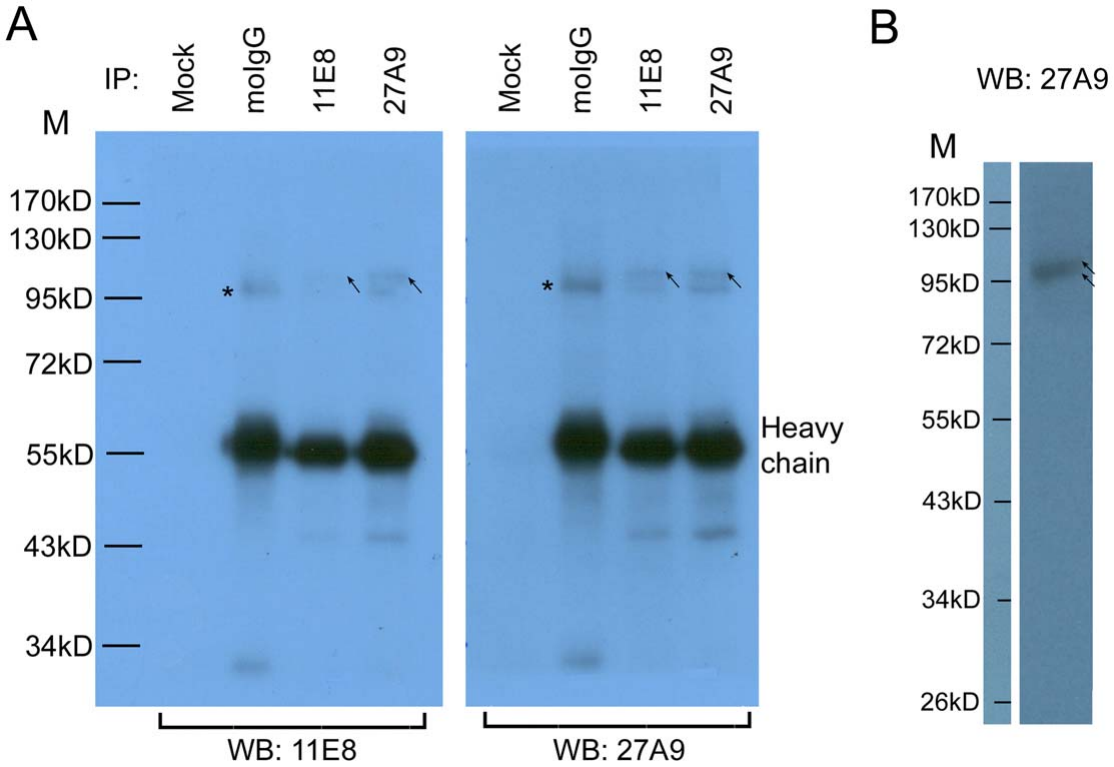
Detection of the native SjAgo2 protein by Western blot. A. Detection of the native SjAgo2 protein in different SWAP immunoprecipitates. Lanes 1 and 5, no antibody added; lanes 2 and 6, immunoprecipitated by normal mouse IgG; lanes 3 and 7, immunoprecipitated by mAb 11E8; lanes 4 and 8, immunoprecipitated by mAb 27A9. Left panel, immunoprecipitates were detected by Western blot with mAb 11E8. Right panel, immunoprecipitates were detected by Western blot with mAb 27A9. HRP-conjugated goat anti-mouse IgG was used as a secondary antibody. The arrows indicate the specific recognition of SjAgo2 by mAbs; the asterisks indicate the band recognized by normal murine IgG. **B.** SWAP was resolved by 12% SDS-PAGE and detected by Western blot with mAb 27A9. The arrows indicate the two bands recognized by mAb 27A9. M, prestained protein ladder SM0671.

Immunoprecipitates from all experimental groups were separated by 10% SDS-PAGE (Figure S4) and followed by Western blot analysis (Figure 3A). Two prominent bands at a molecular weight of approximately 100 kDa were observed. However, the lower band (asterisked) also appeared in the immunoprecipitates captured by normal mouse IgG, indicating that it may have been caused by non-specific binding to mouse IgG. As indicated by the molecular weight, we speculated that the lower band might be the IgG-binding protein paramyosin (PMY) [45]–[47]. In contrast, the upper band (arrowed) is more close to the theoretical molecular weight of SjAgo2 (105.9 kDa). Western blot analysis was performed to determine the reactivity and specificity of the mAb 27A9 directly against SWAP, and two bands (arrowed) with the size of ∼100 kDa were detected (Figure 3B).

By using Orbitrap MS analysis, 38 peptides derived from SjAgo2 were identified from bands between ∼90–130 kDa in 27A9 immunoprecipitates, whereas no peptides derived from SjAgo1 and SjAgo3 were detected in the immunoprecipitates (Table 1), which further confirmed the specificity of the mAb 27A9 to SjAgo2. The RISC forming proteins like TRBP and DDX6 were not identified in the immunoprecipitates. This could be due to the experimental condition which may not be suitable for the coprecipitation of these proteins; or due to the missing sequence information of the two proteins in the *S. japonicum* database which prevented the identification of these two proteins in the MS analysis. The appearance of the 13 peptides derived from PMY in the Orbitrap MS analysis supported our speculation that this was due to its IgG-binding property of the molecule (Table 1). In addition to PMY, several other cytoskeleton and motor proteins, including actin, myosin, dynein, spectrin, and kinesin, were also detected in the immunoprecipitates (Table S2), which were presumably co-purified through interaction with PMY [48]. Strikingly, several members of the heat shock protein (HSP) family (90, 97, and 110 kDa respectively), and three isoforms of the HSP70 protein were identified (Table S2). However, these proteins also appeared in the mock group in the second MS analysis (Table S3), indicating that they were non-specifically captured by the protein-G/A agarose beads. This finding was consistent with the previous observation that the HSP70 homologue in *S. mansoni* (SCHMA-HSP70) can readily bind to protein-G Sepharose [46]. Recent studies in human and flies revealed that HSP90 protein can chaperone Argonautes and facilitate the loading of small RNA duplexes [49]–[51]. More recently, HSP90 was reported to participate in the Piwi-interacting RNA (piRNA) pathway and function in canalization [52]. Our results here suggest that HSP members in *S. japonicum* do not directly interact with SjAgos; thus, whether they can participate in the assembly of RISC complex remains unclear. Nevertheless, as no SjAgo1 and SjAgo3 were detected in 27A9 immunoprecipitates, these co-precipitated contaminating proteins have no influence on analyses of the small RNA population associated with SjAgo2.

**Table 1:** Peptide sequences from SjAgo2 and paramyosin identified by Orbitrap MS from 27A9 immunoprecipitation.

SjAgo2	Paramyosin
R.EYASSSSSMSSR.G	R.KAQQQIEEAEHR.A
F.GADVTHPAPTQNQQIR.K	K.AQQQIEEAEHR.A
R.SSYRPSENIYDKDR.Q	K.HAETELEETQSR.V
R.LSSRPSVSQCPTGELNRR.F	K.YDEESEEASNLR.N
R.YGVVIRQQATTEK.G	R.LKTLTDDLQR.Q
K.SHPIKRPTCDLSVGISK.L	K.SSLESQVDDLKR.S
R.LSSRPSVSQCPTGELNR.R	R.ELEAEFDGESR.R
R.PSVSQCPTGELNR.R	R.LDEAGGSTTQTQELLK.R
R.SQGHIQKVMHELPR.A	K.SAESLASELQR.R
R.RFDEFSR.Q	K.FNADIAALK.S
K.RPTCDLSVGISK.L	R.VKDLETFLDDERR.L
R.QQFIDGPPPSAR.S	R.IQLANEVEELR.S
R.VIHPPSAAFGR.S	R.HQTALNELSLEVENLQK.Q
R.ARNVEPGTIVDTEITHPR.E	
R.SALFYDKPIK.M	
K.RLSDLQMGIR.T	
K.VSTSDVNHFITSPK.A	
R.GPFEQTDAYSSEYDISETARQKK.S	
R.LSDLQMGIR.T	
R.NVEPGTIVDTEITHPR.E	
R.GPFEQTDAYSSEYDISETAR.Q	
R.GPFEQTDAYSSEYDISETARQK.K	
K.MIADKLFTIHK.G	
R.ACADVRPGEEPAITYIVVQKR.H	
R.IMEVQKVSTSDVNHFITSPK.A	
R.PGEEPAITYIVVQKR.H	
R.DQLLLSYR.I	
K.SHPLINQFGLTVQPR.P	
R.SVSYPAPTYYSHLAAFR.A	
R.KSVAAVIGSVSPDLMR.Y	
R.RFTVHGISSVPANQLMIEELK.Q	
R.TLDKPNVFPNLLLK.L	
R.FTVHGISSVPANQLMIEELK.Q	
K.SVAAVIGSVSPDLMR.Y	
R.FQELQTFANNMLK.S	
R.FKVHMSQVDGMFYL.-	
K.VHMSQVDGMFYL.-	
K.LGGVNWQIPDLIK.N	

### Overview of *S. japonicum* small RNA libraries

32,876,012 and 21,822,050 high quality reads were obtained respectively from the two small RNA libraries, SP1 and SP2 (both were established from the SjAgo2 complex with mAb 27A9) (Table S4). The redundancy level of both libraries was ∼85% (Redundancy = 100−(Total Unique Clean Reads/Total High-quality Clean Reads ×100)) (Table S5), which presented a similar sequencing depth as our previous study [18].

**Figure 4:**
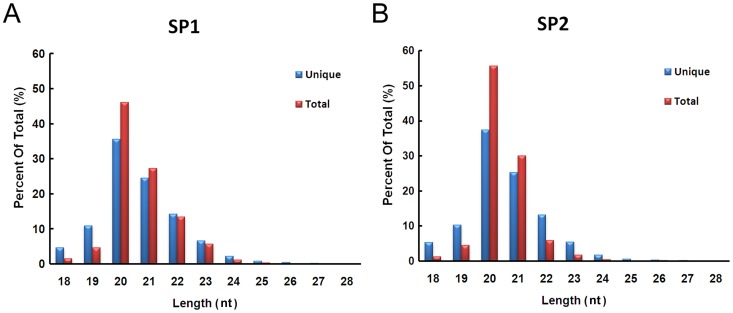
Length distribution of the small RNAs perfectly matched the draft of *S. japonicum* genome. **A.** Length distribution of reads from the SP1 and **B.** SP2 libraries. Both at unique and total levels, the 20-nt reads were predominant in the two libraries, followed by the 21-nt reads.

We investigated the length distribution of small RNA reads in the SP1 and SP2 libraries that perfectly matched the draft genomic sequence of *S. japonicum* (Figure 4). The length distribution of the reads in both libraries presented a quite similar pattern, both at total and unique level. The 20 nt reads were predominant in both libraries, which accounted for 46.1% (SP1) and 55.7% (SP2) of the reads, respectively, followed by the 21 nt reads. Thus, the reads length of sncRNAs associated with SjAgo2 was closer to that of endogenous siRNAs bound to *Drosophila* Ago2, which peaks at 21 nt [25], [53], rather than miRNAs, whose sizes are typically ≈22 nt [54], [55].

### Classification of sncRNAs associated with native SjAgo2 proteins

**Figure 5:**
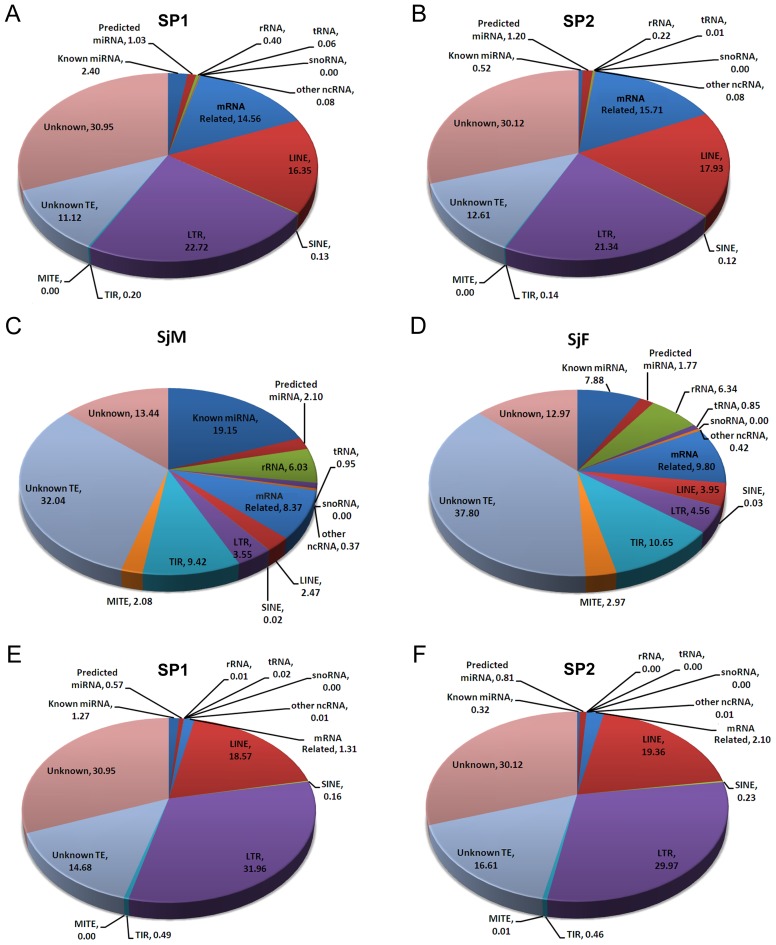
Classification and percentage of small non-coding RNAs in different libraries. **A.** Classification of small RNAs in the SP1 and **B.** SP2 libraries using the bioinformatic pipeline described in [18]. **C.** Small RNA classification of the SjM and **D.** SjF libraries using the data from our previous study [18]. **E.** Small RNA classification of the SP1 and **F.** SP2 libraries using an alternative bioinformatic pipeline as described in the Materials and Methods.

We systematically defined the sncRNAs in both libraries SP1 and SP2 (Figure 5A and B), using the bioinformatic pipeline as reported previously [18]. We also compared the data to that obtained from the adult worm libraries SjM and SjF, which were constructed with total small RNA (Figure 5C and D) [18]. The proportions of LTR- and LINE-derived siRNAs were significantly higher than that of miRNA, rRNA, TIR- and MITE-derived siRNAs in the two libraries compared to that constructed with total small RNAs. For the LINE-derived siRNAs, the proportion increased from ≈3% in the adult small RNA libraries to an average of 17% in the SjAgo2-specific libraries. For the LTR-derived siRNAs, the proportion in the SjAgo2-specific libraries was at least 5-fold higher than that in the libraries SjF and SjM (from ≈4% to an average of 22%). This difference strongly suggests that SjAgo2 preferentially associated with siRNAs derived from LINE and LTR retrotransposons.

Regarding the mRNA related small RNAs, the proportion of this group in SjAgo2-specific libraries was twice as high as that in the small RNA libraries of separated adult worms (Figure 5A, B, C, and D). This is due to the reason that numerous TE-derived transcripts were deposited in the predicted *S. japonicum* database as mRNA sequences (sjr_mRNA.fasta). Thus, a mass of TE-derived siRNAs may have been categorized as mRNA-related small RNAs. Therefore, an optimized bioinformatic pipeline was designed to sort the small RNAs from SjAgo2-specific libraries. As a result, the proportion of mRNA-related small RNAs substantially decreased in contrast to that of retrotransposon-derived siRNAs, in particular LTR-derived siRNAs, which increased nearly one-third (Figure 5E and F). This observation further implies that SjAgo2 predominantly interacts with retrotransposon-derived siRNAs.

### SiRNAs interacted with SjAgo2 were restricted to several classes of retrotransposons

**Figure 6:**
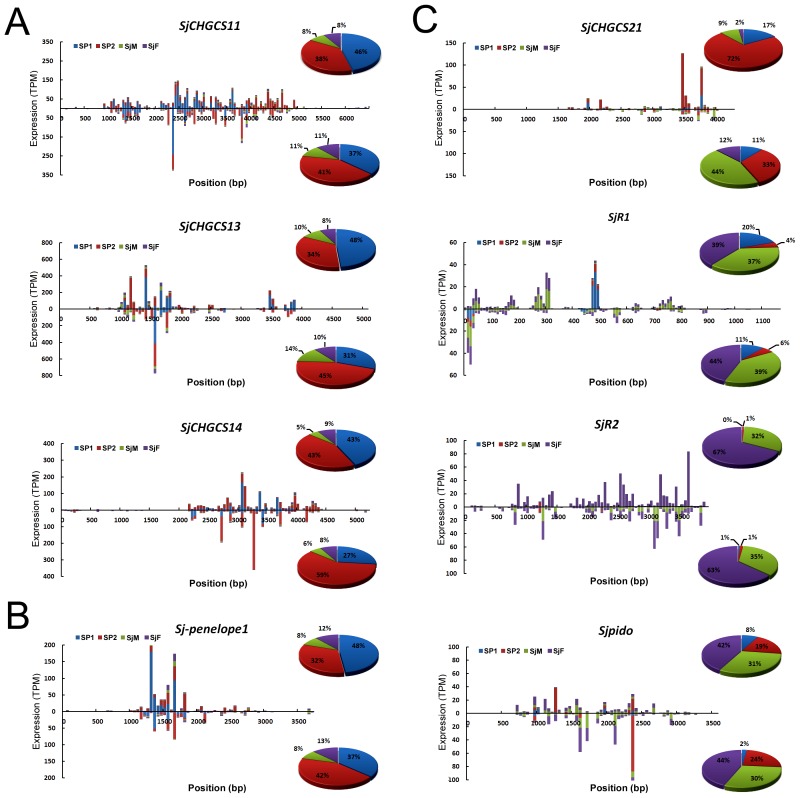
The distribution and abundance of retrotransposon-derived siRNAs in different small RNA libraries from adult worms. Bars with different colors were created to indicate the abundance (reflected as TPM value) of retrotransposon-derived siRNAs in different libraries. **A.** Endo-siRNAs mapped to the LTR retrotransposons, *SjCHGCS11*, *SjCHGCS13*, and *SjCHGCS14*. Both sense and antisense siRNAs generated from these LTR elements were enriched in the SjAgo2-specific libraries SP1 and SP2. **B.** Endo-siRNAs mapped to *Penelope*-like retrotransposon *Sj-penelope1*. Both sense and antisense siRNAs generated from the retrotransposon were dramatically accumulated in the SP1 and SP2 libraries. **C.** Endo-siRNAs mapped to the LINEs, *SjCHGCS21*, *SjR1*, *SjR2*, and *Sjpid*o. Sense siRNAs originated from SjCH*GCS21* were enriched in the SjAgo2-specific libraries. However, siRNAs generated from *SjR1* and *Sjpido* were at a low level in the SP1 and SP2 libraries. *SjR2*-derived siRNAs were hardly detected in these two libraries. The percentage of total siRNAs derived from a particular TE among the four libraries was calculated by using the sum of TPM value of each siRNA and was displayed in the pie charts. The upper panel in each chart represents sense siRNAs; the lower panel in each chart represents antisense siRNAs.

TE components have been recognized as one of the principal forces driving genome diversity and evolution [56]. However, too many insertions of TEs into the genome may be deleterious, imposing that they must be under appropriate control to keep the integrity of the genome [57]. In *S. japonicum*, the repetitive elements account for more than 40% of the genome sequences [4]. And the mobile genetic elements (MGEs) in *S. japonicum* have been categorized into several classes, including short interspersed nucleotide elements (SINEs)-like retrotransposons [58], LTR [4], [41], non-LTR [4], [42], and *Penelope*-like retrotransposons [4]. We therefore further investigated whether the small RNAs interacted with SjAgo2 were restricted to any particular class of retrotransposons. The expression levels of siRNAs derived from 29 well-defined retrotransposons in the SP1, SP2, SjM, and SjF libraries were presented based on their TPM value (Table 2). We found that siRNAs in the SjAgo2-specific libraries were mainly derived from 11 classes of retrotransposons (Table 2, Top 11). For example, siRNAs generated from retrotransposon *SjCHGCS11*, *SjCHGCS13*, *SjCHGCS14*, and *Sj-penelope1* were 4–6 fold more in the SjAgo2-specific libraries than that in the libraries SjM and SjF (Figure 6A and B). Sense siRNAs generated from LINE *SjCHGCS21* were also enriched in the SP1 and SP2 libraries (Figure 6C). In contrast, the abundance of siRNAs derived from *SjCHGCS10*, *Sjpido*, *SjCHGCS1*, *SjCHGCS2*, *SjCHGCS19*, *SjCHGCS20*, *SjR2*, and *SjCHGCS3* was decreased in the SjAgo2-specific libraries compared to that in the SjM and SjF libraries (Table 2, and Figure 6C), suggesting that the function of siRNAs from these classes of retrotransposons were correlated to SjAgo2.

**Table 2:** Transcriptional levels (TPM) of 29 types of well-defined retrotransposons and the corresponding siRNAs in different libraries from adult worms of *S. japonicum*.

TE types	GenBank Accession number	SiRNAs								Transcripts	
		Sense				Antisense				Total distinct tag	
		SP1	SP2	SjM	SjF	SP1	SP2	SjM	SjF	Sj-M^1^	Sj-F^2^
*SjCHGCS13*	FN356215	4147.0	2971.7	876.4	705.1	2004.4	3068.6	942.8	727.8	230.9	208.6
*SjCHGCS11*	FN356213	3504.0	2904.6	618.0	665.0	2542.0	2813.3	746.8	815.9	182.8	437.5
*SjCHGCS6*	FN356208	1598.4	2230.9	237.2	268.7	576.2	2521.7	251.5	290.7	48.4	58.1
*SjCHGCS14*	FN356216	1968.5	1972.1	224.5	418.8	1115.9	2538.4	236.9	356.7	170.8	277.3
*Sj-penelope1*	FN356225	980.2	682.0	174.8	249.4	457.6	540.6	103.9	161.5	23.7	20.9
*Sj-penelope2*	FN356226	257.2	1012.9	130.4	171.0	333.3	91.9	132.1	201.3	43.0	28.5
*SjCHGCS21*	FN356223	169.0	728.6	97.7	24.3	50.8	157.2	221.6	59.9	110.2	126.4
*SjCHGCS9*	FN356211	257.9	363.1	127.1	152.7	237.7	573.3	64.3	94.7	0.7	1.8
*SjCHGCS4*	FN356206	520.6	57.1	106.3	136.1	588.9	3157.2	125.7	216.3	23.2	19.7
*SjCHGCS16*	FN356218	428.3	549.6	635.0	819.2	1056.7	2759.3	339.7	442.3	898.6	427.9
*SjCHGCS7*	FN356209	555.9	976.4	400.2	810.4	232.1	349.8	169.2	279.0	32.5	90.6
*SjCHGCS5*	FN356207	42.0	31.3	27.8	43.0	3.3	15.0	30.8	43.2	258.7	279.6
*SjCHGCS18*	FN356220	2.2	0.2	0.4	0.4	2.0	0.2	0.0	0.1	11.2	13.3
*Sj-penelope3*	FN356227	1.2	0.0	0.6	0.9	2.4	0.2	4.2	7.1	0.7	0.8
*SjCHGCS22*	FN356224	0.0	0.2	1.3	3.6	0.0	0.0	0.4	0.3	13.6	11.9
*Gulliver*	AF243513	0.0	0.0	2.7	7.1	0.0	0.0	0.8	1.3	9.7	8.4
*SjR1*	AF073333	34.1	7.7	69.8	74.9	14.3	7.0	54.2	60.6	111.6	59.7
*SjCHGCS8*	FN356210	40.5	14.1	48.8	120.6	10.6	12.1	9.1	24.9	0.2	12.1
*SjCHGCS15*	FN356217	125.9	96.9	190.0	206.8	130.2	250.6	251.8	259.5	142.6	195.1
*SjCHGCS12*	FN356214	306.5	80.1	219.1	410.9	60.4	83.4	351.8	493.5	303.3	173.2
*SjCHGCS17*	FN356219	33.4	73.4	170.9	192.4	1536.6	348.7	651.5	585.6	202.3	89.0
*SjCHGCS10*	FN356212	5.6	27.4	108.8	226.9	16.4	87.1	98.1	164.4	175.7	123.8
*Sjpido*	AY034003	72.9	183.4	235.7	363.7	32.4	247.4	319.2	483.9	376.9	252.0
*SjCHGCS1*	FN356203	23.8	25.9	157.0	314.8	73.5	9.9	169.8	329.4	30.8	27.5
*SjCHGCS2*	FN356204	17.1	131.2	245.1	432.7	35.0	133.7	297.5	474.5	176.9	92.2
*SjCHGCS19*	FN356221	12.9	7.0	510.0	885.9	18.4	9.4	245.5	600.8	172.6	190.3
*SjCHGCS20*	FN356222	25.4	14.9	923.6	1369.1	4.6	287.3	895.0	1204.2	647.2	655.4
*SjR2*	AY027869	2.7	17.8	829.9	1741.0	13.3	24.7	612.9	1106.2	859.7	716.7
*SjCHGCS3*	FN356205	380.1	127.0	1571.3	2680.5	177.1	291.9	591.4	1406.4	681.1	439.7

1 Whole-transcriptome RNA-seq library from male adult worm of *S. japonicum*.

2 Whole-transcriptome RNA-seq library from female adult worm of *S. japonicum*.

### The potential function of *S. japonicum* Argonaute proteins

We further evaluated the correlation between the transcription levels of the well-defined retrotransposons of *S. japonicum* and the enrichment of the siRNAs in the SjAgo2 complex by analysis of the whole-transcriptome data generated from separated adult worms (Piao *et al.*, unpublished data). Interestingly, for several classes of retrotransposons, an obvious inverse relationship was observed between the abundance of mRNA transcripts and amount of relevant siRNAs in the SjAgo2-specific libraries. For instance, siRNAs derived from retrotransposon *SjCHGCS6*, *Sj-penelop*e*1*, *Sj-penelope2*, *SjCHGCS21*, *SjCHGCS9*, and *SjCHGCS4* were highly enriched in the SjAgo2 libraries, whereas the levels of the corresponding transcripts of these mobile elements were much lower (Table 2). On the contrary, siRNAs derived from retrotransposon *SjCHGCS20*, *SjR2*, and *SjCHGCS3* were much less in the SjAgo2 libraries, the transcripts of these retroelements were relatively more (Table 2). These findings suggest that siRNAs enriched in the SjAgo2 libraries were not affected by the transcription levels of the retrotransposons, and SjAgo2 may be functionally specialized to suppress a group of transposable elements in the parasite. However, this regulatory model cannot be applied to all types of retrotransposons. It can be explained by the facts that the transcriptome data reflect the transcriptional levels of retroelements within the whole worms, while the expression of SjAgo2 in the parasite may be tissue-specific as its ortholog in *S. mansoni* [59].

Based on the property of its associated small RNA population, we postulated that SjAgo2 is mainly involved in such a mechanism by regulating retrotransposon at the transcriptional level. A similar function of Argonaute protein has previously been suggested in studies of *Trypanosoma brucei*, *D. melanogaster*, and mice [25], [53], [60]–[63]. In addition, the Ago2 transcripts in *S. mansoni* exhibited a germline-specific expression in both adult female and male worms [59]. This observation indicates that, in schistosome adult worms, Ago2 functions in the maintenance of genome stability in germline cells by retrotransposons silencing. Previous studies in *Drosophila* and vertebrates have shown that the endo-siRNA pathway is involved in transposons silencing in somatic tissues [25], [53], [57], [60], [63]; whereas transposons are mainly controlled by the piRNA pathway in germline cells, which functions through Piwi subclade proteins [64], [65]. However, the piRNA pathway does not appear to be specialized in schistosome as no Piwi homolog has been discovered in its genome [15], [31]. The siRNA pathway mediated by SjAgo2 in schistosome germline could, to some extent, compensate for the absence of the piRNA pathway as suggested previously [31]. Given the fact that SjAgo2 is ubiquitously expressed during various developmental stages of the parasite, though at different levels, SjAgo2 may be bi-functional in both somatic and germline cells. However, further studies are needed to dissect it out.

Though the PAZ and Piwi domains were highly homologous between SjAgo2 and SjAgo3, substantial differences exist in the region corresponding to the typical Mid domain, which has been definitively established to play role in 5′ end recognition of the guide strand [27], [66]. The reverse expression pattern of *SjAgo2* and *SjAgo3* genes in male and female adult worms was also observed (Figure 1). Both of these observations indicate that SjAgo3 may play an analogous, but non-redundant role to SjAgo2 in *S. japonicum*, such as suppressing the activities of TEs in somatic cells. One line of evidence supporting this is that a substantial portion of small RNAs derived from DNA transposons TIR and MITE was detected in adult worms, with an amount even more than that of LTR- and LINE-derived siRNAs in both male and female worms (Figure 5C and D). Another possibility is that SjAgo3 may also restrict the activities of retrotransposons, such as *SjR1*, *SjR2*, and *Sjpido*, via binding with siRNAs that not enriched in the SjAgo2-specific libraries (Figure 6C).

Only a small proportion of miRNAs was found to be associated with SjAgo2 (Figure 5A and B), which is in line with the suggestion that the miRNA pathway in schistosomes is mainly mediated by Drosha, Dicer, and Ago1, as Ago1 is more closely related to Argonaute orthologs involved in the miRNA pathway in flies, humans, and worms [31]. Our findings here as well as those found in *Drosophila* suggest that some miRNAs were still bound to their unconventional partner, Ago2, in addition to being strongly associated with Ago1 [67], [68]. Thus, the phenomenon that some miRNAs sorted onto the SjAgo2 complex exhibits the complexity of a small RNA regulatory network in schistosome parasite and suggests that different silencing pathways may cross-link with each other and share or compete the apparatus required in the biogenesis of different small RNAs. In *Drosophila*, miRNA are generated in a Dicer1-dependent manner, whereas siRNAs are produced in a Dicer2-dependently manner [60]. However, the dsRNA-binding protein Loquacious (Loqs), a typical miRNA factor associated with Dicer1, may actually be required for the biogenesis of endo-siRNAs [25], [69]. Since only one Dicer gene was found in *S. japonicum* [28], the miRNA pathway and endo-siRNA system in schistosomes may share one Dicer in the production of miRNA and siRNA duplex, cross-linking both pathways at upstream.

In summary, using a mAb specific to SjAgo2, we have systematically investigated the small RNAs bound to the protein. SjAgo2 was determined to associate mainly with endo-siRNAs derived from LINE and LTR types of transposable elements in adult *S. japonicum*. The enrichment of siRNAs in the SjAgo2-specific libraries was found to be restricted to particular types of retrotransposons. These results emphasize the potential role of SjAgo2 in maintaining genomic stability in germ-line and/or somatic cells by repressing retrotransposons.

## Supporting Information

Figure S1 **SWAP immunoprecipitates were resolved on 10% SDS-PAGE. Sequential IP assays were carried out as described in the** **Materials and Methods** **.** Protein bands with different molecular weights located in the squares were excised from SDS-PAGE gel and anylzed by MS.
(TIF)

Figure S2 **Agilent 2100 Bioanalyzer analysis of small RNA samples co-precipitated with SjAgo2 by two 27A9 IP assays.** The predominant species of the small RNAs was around 25 nt.
(TIF)

Figure S3 **The expression of Flag-tagged SjAgos in 293T cells was detected by Western blot with mAb M2 (anti-Flag) after adjusting the loading volumes of protein samples.**
(TIF)

Figure S4 **SWAP was sequentially incubated with pure Protein-A/G agarose beads only (Mock), and normal mouse IgG, mAb 11E8, and 27A9.** The precipitated protein complexes were resolved on 10% SDS-PAGE, stained with Coomasie brilliant blue.
(TIF)

Table S1 **Sequences of the primers used for PCR experiments.**
(XLS)

Table S2 **Proteins identified by Orbitrap MS in immunoprecipitation with mAb 27A9.**
(XLS)

Table S3 **Proteins identified by Orbitrap MS in different immunoprecipitates.**
(XLS)

Table S4 **General information of the two small RNA libraries (SP1 and SP2).**
(XLS)

Table S5 **Data statistics of the two small RNA libraries (SP1 and SP2).**
(XLS)

## References

[1] Gobert GN, Moertel L, Brindley PJ, McManus DP (2009) Developmental gene expression profiles of the human pathogen *Schistosoma japonicum* . BMC Genomics 10: 128. 19320991 10.1186/1471-2164-10-128 PMC2670322

[2] Blanchard TJ (2004) Schistosomiasis. Travel Med Infect Dis 2: 5–11. 17291950 10.1016/j.tmaid.2004.02.011

[3] Liu F, Lu J, Hu W, Wang SY, Cui SJ, et al (2006) New perspectives on host-parasite interplay by comparative transcriptomic and proteomic analyses of *Schistosoma japonicum* . PLoS Pathog 2: e29. 16617374 10.1371/journal.ppat.0020029 PMC1435792

[4] Zhou Y, Zheng HJ, Chen YY, Zhang L, Wang K, et al (2009) The *Schistosoma japonicum* genome reveals features of host-parasite interplay. Nature 460: 345–351. 19606140 10.1038/nature08140 PMC3747554

[5] Gobert GN, Tran MH, Moertel L, Mulvenna J, Jones MK, et al (2010) Transcriptional changes in *Schistosoma mansoni* during early schistosomula development and in the presence of erythrocytes. PLoS Negl Trop Dis 4: e600. 20161728 10.1371/journal.pntd.0000600 PMC2817720

[6] Piao X, Cai P, Liu S, Hou N, Hao L, et al (2011) Global expression analysis revealed novel gender-specific gene expression features in the blood fluke parasite *Schistosoma japonicum* . PLoS One 6: e18267. 21494327 10.1371/journal.pone.0018267 PMC3071802

[7] Berriman M, Haas BJ, LoVerde PT, Wilson RA, Dillon GP, et al (2009) The genome of the blood fluke *Schistosoma mansoni* . Nature 460: 352–358. 19606141 10.1038/nature08160 PMC2756445

[8] Young ND, Jex AR, Li B, Liu S, Yang L, et al (2012) Whole-genome sequence of *Schistosoma haematobium* . Nat Genet 44: 221–225. 22246508 10.1038/ng.1065

[9] Pfeffer S, Sewer A, Lagos-Quintana M, Sheridan R, Sander C, et al (2005) Identification of microRNAs of the herpesvirus family. Nat Methods 2: 269–276. 15782219 10.1038/nmeth746

[10] Brennecke J, Aravin AA, Stark A, Dus M, Kellis M, et al (2007) Discrete small RNA-generating loci as master regulators of transposon activity in *Drosophila* . Cell 128: 1089–1103. 17346786 10.1016/j.cell.2007.01.043

[11] Bartel DP (2009) MicroRNAs: target recognition and regulatory functions. Cell 136: 215–233. 19167326 10.1016/j.cell.2009.01.002 PMC3794896

[12] Molnar A, Melnyk CW, Bassett A, Hardcastle TJ, Dunn R, et al (2010) Small Silencing RNAs in Plants Are Mobile and Direct Epigenetic Modification in Recipient Cells. Science 328: 872–875. 20413459 10.1126/science.1187959

[13] Xue X, Sun J, Zhang Q, Wang Z, Huang Y, et al (2008) Identification and characterization of novel microRNAs from *Schistosoma japonicum* . PLoS One 3: e4034. 19107204 10.1371/journal.pone.0004034 PMC2603315

[14] Huang J, Hao P, Chen H, Hu W, Yan Q, et al (2009) Genome-wide identification of *Schistosoma japonicum* microRNAs using a deep-sequencing approach. PLoS One 4: e8206. 19997615 10.1371/journal.pone.0008206 PMC2785426

[15] Hao L, Cai P, Jiang N, Wang H, Chen Q (2010) Identification and characterization of microRNAs and endogenous siRNAs in *Schistosoma japonicum* . BMC Genomics 11: 55. 20092619 10.1186/1471-2164-11-55 PMC2820009

[16] Wang Z, Xue X, Sun J, Luo R, Xu X, et al (2010) An in-depth description of the small non-coding RNA population of *Schistosoma japonicum* schistosomulum. PLoS Negl Trop Dis 4: e596. 20161724 10.1371/journal.pntd.0000596 PMC2817716

[17] de Souza Gomes M, Muniyappa MK, Carvalho SG, Guerra-Sa R, Spillane C (2011) Genome-wide identification of novel microRNAs and their target genes in the human parasite *Schistosoma mansoni* . Genomics 98: 96–111. 21640815 10.1016/j.ygeno.2011.05.007

[18] Cai P, Hou N, Piao X, Liu S, Liu H, et al (2011) Profiles of small non-coding RNAs in *Schistosoma japonicum* during development. PLoS Negl Trop Dis 5: e1256. 21829742 10.1371/journal.pntd.0001256 PMC3149011

[19] Simoes MC, Lee J, Djikeng A, Cerqueira GC, Zerlotini A, et al (2011) Identification of *Schistosoma mansoni* microRNAs. BMC Genomics 12: 47. 21247453 10.1186/1471-2164-12-47 PMC3034697

[20] Hutvagner G, Simard MJ (2008) Argonaute proteins: key players in RNA silencing. Nat Rev Mol Cell Biol 9: 22–32. 18073770 10.1038/nrm2321

[21] Yigit E, Batista PJ, Bei Y, Pang KM, Chen CC, et al (2006) Analysis of the *C. elegans* Argonaute family reveals that distinct Argonautes act sequentially during RNAi. Cell 127: 747–757. 17110334 10.1016/j.cell.2006.09.033

[22] Braun L, Cannella D, Ortet P, Barakat M, Sautel CF, et al (2010) A complex small RNA repertoire is generated by a plant/fungal-like machinery and effected by a metazoan-like Argonaute in the single-cell human parasite *Toxoplasma gondii* . PLoS Pathog 6: e1000920. 20523899 10.1371/journal.ppat.1000920 PMC2877743

[23] Allen E, Xie Z, Gustafson AM, Carrington JC (2005) microRNA-directed phasing during trans-acting siRNA biogenesis in plants. Cell 121: 207–221. 15851028 10.1016/j.cell.2005.04.004

[24] Das PP, Bagijn MP, Goldstein LD, Woolford JR, Lehrbach NJ, et al (2008) Piwi and piRNAs act upstream of an endogenous siRNA pathway to suppress Tc3 transposon mobility in the *Caenorhabditis elegans* germline. Mol Cell 31: 79–90. 18571451 10.1016/j.molcel.2008.06.003 PMC3353317

[25] Chung WJ, Okamura K, Martin R, Lai EC (2008) Endogenous RNA interference provides a somatic defense against *Drosophila* transposons. Curr Biol 18: 795–802. 18501606 10.1016/j.cub.2008.05.006 PMC2812477

[26] Gomes MS, Cabral FJ, Jannotti-Passos LK, Carvalho O, Rodrigues V, et al (2009) Preliminary analysis of miRNA pathway in *Schistosoma mansoni* . Parasitol Int 58: 61–68. 19007911 10.1016/j.parint.2008.10.002

[27] Chen J, Yang Y, Guo S, Peng J, Liu Z, et al (2010) Molecular cloning and expression profiles of Argonaute proteins in *Schistosoma japonicum* . Parasitol Res 107: 889–899. 20582438 10.1007/s00436-010-1946-3

[28] Luo R, Xue X, Wang Z, Sun J, Zou Y, et al (2010) Analysis and characterization of the genes encoding the Dicer and Argonaute proteins of *Schistosoma japonicum* . Parasit Vectors 3: 90. 20849617 10.1186/1756-3305-3-90 PMC2949827

[29] Reddien PW, Oviedo NJ, Jennings JR, Jenkin JC, Sanchez Alvarado A (2005) SMEDWI-2 is a PIWI-like protein that regulates planarian stem cells. Science 310: 1327–1330. 16311336 10.1126/science.1116110

[30] Palakodeti D, Smielewska M, Lu YC, Yeo GW, Graveley BR (2008) The PIWI proteins SMEDWI-2 and SMEDWI-3 are required for stem cell function and piRNA expression in planarians. Rna 14: 1174–1186. 18456843 10.1261/rna.1085008 PMC2390803

[31] Batista TM, Marques JT (2011) RNAi pathways in parasitic protists and worms. J Proteomics 74: 1504–1514. 21385631 10.1016/j.jprot.2011.02.032

[32] Dalton JP, Day SR, Drew AC, Brindley PJ (1997) A method for the isolation of schistosome eggs and miracidia free of contaminating host tissues. Parasitology 115 (Pt 1) 29–32. 9226954 10.1017/s0031182097001091

[33] Liu S, Cai P, Hou N, Piao X, Wang H, et al (2012) Genome-wide identification and characterization of a panel of house-keeping genes in *Schistosoma japonicum* . Mol Biochem Parasitol 182: 75–82. 22245333 10.1016/j.molbiopara.2011.12.007

[34] Maniataki E, De Planell Saguer MD, Mourelatos Z (2005) Immunoprecipitation of microRNPs and directional cloning of microRNAs. Methods Mol Biol 309: 283–294. 15990408 10.1385/1-59259-935-4:283

[35] Cai P, Bu L, Wang J, Wang Z, Zhong X, et al (2008) Molecular characterization of *Schistosoma japonicum* tegument protein tetraspanin-2: sequence variation and possible implications for immune evasion. Biochem Biophys Res Commun 372: 197–202. 18486598 10.1016/j.bbrc.2008.05.042

[36] Li R, Yu C, Li Y, Lam TW, Yiu SM, et al (2009) SOAP2: an improved ultrafast tool for short read alignment. Bioinformatics 25: 1966–1967. 19497933 10.1093/bioinformatics/btp336

[37] Griffiths-Jones S, Grocock RJ, van Dongen S, Bateman A, Enright AJ (2006) miRBase: microRNA sequences, targets and gene nomenclature. Nucleic Acids Res 34: D140–144. 16381832 10.1093/nar/gkj112 PMC1347474

[38] Griffiths-Jones S, Saini HK, van Dongen S, Enright AJ (2008) miRBase: tools for microRNA genomics. Nucleic Acids Res 36: D154–158. 17991681 10.1093/nar/gkm952 PMC2238936

[39] Dsouza M, Larsen N, Overbeek R (1997) Searching for patterns in genomic data. Trends Genet 13: 497–498. 9433140 10.1016/s0168-9525(97)01347-4

[40] Griffiths-Jones S, Moxon S, Marshall M, Khanna A, Eddy SR, et al (2005) Rfam: annotating non-coding RNAs in complete genomes. Nucleic Acids Res 33: D121–124. 15608160 10.1093/nar/gki081 PMC540035

[41] Laha T, Loukas A, Verity CK, McManus DP, Brindley PJ (2001) *Gulliver*, a long terminal repeat retrotransposon from the genome of the oriental blood fluke *Schistosoma japonicum* . Gene 264: 59–68. 11245979 10.1016/s0378-1119(00)00601-6

[42] Laha T, Brindley PJ, Verity CK, McManus DP, Loukas A (2002) *pido*, a non-long terminal repeat retrotransposon of the chicken repeat 1 family from the genome of the Oriental blood fluke, *Schistosoma japonicum* . Gene 284: 149–159. 11891056 10.1016/s0378-1119(02)00381-5

[43] Laha T, Brindley PJ, Smout MJ, Verity CK, McManus DP, et al (2002) Reverse transcriptase activity and untranslated region sharing of a new RTE-like, non-long terminal repeat retrotransposon from the human blood fluke, *Schistosoma japonicum* . Int J Parasitol 32: 1163–1174. 12117499 10.1016/s0020-7519(02)00063-2

[44] Benson DA, Karsch-Mizrachi I, Clark K, Lipman DJ, Ostell J, et al (2012) GenBank. Nucleic Acids Res 40: D48–53. 22144687 10.1093/nar/gkr1202 PMC3245039

[45] Loukas A, Jones MK, King LT, Brindley PJ, McManus DP (2001) Receptor for Fc on the surfaces of schistosomes. Infect Immun 69: 3646–3651. 11349025 10.1128/IAI.69.6.3646-3651.2001 PMC98357

[46] McIntosh RS, Jones FM, Dunne DW, McKerrow JH, Pleass RJ (2006) Characterization of immunoglobulin binding by schistosomes. Parasite Immunol 28: 407–419. 16916364 10.1111/j.1365-3024.2006.00829.x

[47] Wu C, Cai P, Chang Q, Hao L, Peng S, et al (2011) Mapping the binding between the tetraspanin molecule (Sjc23) of *Schistosoma japonicum* and human non-immune IgG. PLoS One 6: e19112. 21533061 10.1371/journal.pone.0019112 PMC3080413

[48] Jones MK, Gobert GN, Zhang L, Sunderland P, McManus DP (2004) The cytoskeleton and motor proteins of human schistosomes and their roles in surface maintenance and host-parasite interactions. Bioessays 26: 752–765. 15221857 10.1002/bies.20058

[49] Johnston M, Geoffroy MC, Sobala A, Hay R, Hutvagner G (2010) HSP90 protein stabilizes unloaded argonaute complexes and microscopic P-bodies in human cells. Mol Biol Cell 21: 1462–1469. 20237157 10.1091/mbc.E09-10-0885 PMC2861606

[50] Iwasaki S, Kobayashi M, Yoda M, Sakaguchi Y, Katsuma S, et al (2010) Hsc70/Hsp90 chaperone machinery mediates ATP-dependent RISC loading of small RNA duplexes. Mol Cell 39: 292–299. 20605501 10.1016/j.molcel.2010.05.015

[51] Miyoshi T, Takeuchi A, Siomi H, Siomi MC (2010) A direct role for Hsp90 in pre-RISC formation in *Drosophila* . Nat Struct Mol Biol 17: 1024–1026. 20639883 10.1038/nsmb.1875

[52] Gangaraju VK, Yin H, Weiner MM, Wang J, Huang XA, et al (2011) *Drosophila* Piwi functions in Hsp90-mediated suppression of phenotypic variation. Nat Genet 43: 153–158. 21186352 10.1038/ng.743 PMC3443399

[53] Kawamura Y, Saito K, Kin T, Ono Y, Asai K, et al (2008) *Drosophila* endogenous small RNAs bind to Argonaute 2 in somatic cells. Nature 453: 793–797. 18463636 10.1038/nature06938

[54] Okamura K, Balla S, Martin R, Liu N, Lai EC (2008) Two distinct mechanisms generate endogenous siRNAs from bidirectional transcription in *Drosophila melanogaster* . Nat Struct Mol Biol 15: 581–590. 18500351 10.1038/nsmb.1438 PMC2713754

[55] Chen X, Li Q, Wang J, Guo X, Jiang X, et al (2009) Identification and characterization of novel amphioxus microRNAs by Solexa sequencing. Genome Biol 10: R78. 19615057 10.1186/gb-2009-10-7-r78 PMC2728532

[56] Biemont C, Vieira C (2006) Genetics: junk DNA as an evolutionary force. Nature 443: 521–524. 17024082 10.1038/443521a

[57] Obbard DJ, Finnegan DJ (2008) RNA interference: endogenous siRNAs derived from transposable elements. Curr Biol 18: R561–563. 18606126 10.1016/j.cub.2008.05.035

[58] Laha T, McManus DP, Loukas A, Brindley PJ (2000) Sjalpha elements, short interspersed element-like retroposons bearing a hammerhead ribozyme motif from the genome of the oriental blood fluke *Schistosoma japonicum* . Biochim Biophys Acta 1492: 477–482. 11004517 10.1016/s0167-4781(00)00112-3

[59] Cogswell AA, Collins JJ 3rd, Newmark PA, Williams DL (2011) Whole mount in situ hybridization methodology for *Schistosoma mansoni* . Mol Biochem Parasitol 178: 46–50. 21397637 10.1016/j.molbiopara.2011.03.001 PMC3102561

[60] Ghildiyal M, Seitz H, Horwich MD, Li C, Du T, et al (2008) Endogenous siRNAs derived from transposons and mRNAs in *Drosophila* somatic cells. Science 320: 1077–1081. 18403677 10.1126/science.1157396 PMC2953241

[61] Watanabe T, Totoki Y, Toyoda A, Kaneda M, Kuramochi-Miyagawa S, et al (2008) Endogenous siRNAs from naturally formed dsRNAs regulate transcripts in mouse oocytes. Nature 453: 539–543. 18404146 10.1038/nature06908

[62] Shi H, Djikeng A, Tschudi C, Ullu E (2004) Argonaute protein in the early divergent eukaryote *Trypanosoma brucei*: control of small interfering RNA accumulation and retroposon transcript abundance. Mol Cell Biol 24: 420–427. 14673174 10.1128/MCB.24.1.420-427.2004 PMC303348

[63] Czech B, Malone CD, Zhou R, Stark A, Schlingeheyde C, et al (2008) An endogenous small interfering RNA pathway in *Drosophila* . Nature 453: 798–802. 18463631 10.1038/nature07007 PMC2895258

[64] Vagin VV, Sigova A, Li C, Seitz H, Gvozdev V, et al (2006) A distinct small RNA pathway silences selfish genetic elements in the germline. Science 313: 320–324. 16809489 10.1126/science.1129333

[65] Houwing S, Kamminga LM, Berezikov E, Cronembold D, Girard A, et al (2007) A role for Piwi and piRNAs in germ cell maintenance and transposon silencing in Zebrafish. Cell 129: 69–82. 17418787 10.1016/j.cell.2007.03.026

[66] Yuan YR, Pei Y, Ma JB, Kuryavyi V, Zhadina M, et al (2005) Crystal structure of *A. aeolicus* argonaute, a site-specific DNA-guided endoribonuclease, provides insights into RISC-mediated mRNA cleavage. Mol Cell 19: 405–419. 16061186 10.1016/j.molcel.2005.07.011 PMC4689305

[67] Forstemann K, Horwich MD, Wee L, Tomari Y, Zamore PD (2007) *Drosophila* microRNAs are sorted into functionally distinct argonaute complexes after production by dicer-1. Cell 130: 287–297. 17662943 10.1016/j.cell.2007.05.056 PMC2686109

[68] Siomi MC, Saito K, Siomi H (2008) How selfish retrotransposons are silenced in *Drosophila* germline and somatic cells. FEBS Lett 582: 2473–2478. 18572018 10.1016/j.febslet.2008.06.018

[69] Ghildiyal M, Zamore PD (2009) Small silencing RNAs: an expanding universe. Nat Rev Genet 10: 94–108. 19148191 10.1038/nrg2504 PMC2724769

