# Supplementary figures and images for: Identification and Characterization of Argonaute Protein, Ago2 and Its Associated Small RNAs in Schistosoma japonicum
Source: PLoS Negl Trop Dis. 2012 Jul 31;6(7):e1745. doi: 10.1371/journal.pntd.0001745 (PMC3409120; doi:10.1371/journal.pntd.0001745)

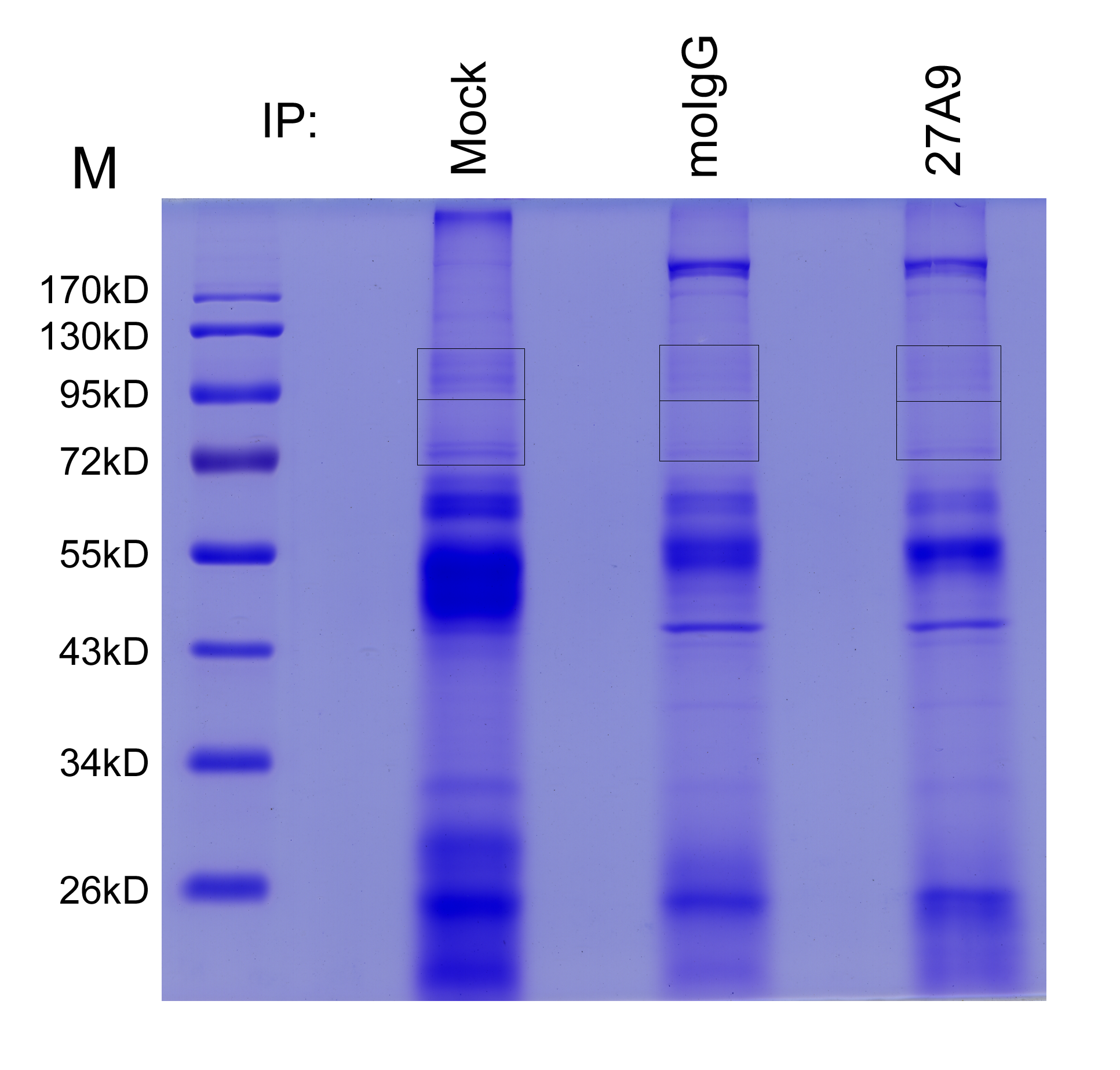

Supplement: Figure S1 — SWAP immunoprecipitates were resolved on 10% SDS-PAGE. Sequential IP assays were carried out as described in the Materials and Methods . Protein bands with different molecular weights located in the squares were excised from SDS-PAGE gel and anylzed by MS. (TIF) [file pntd.0001745.s001.tif]

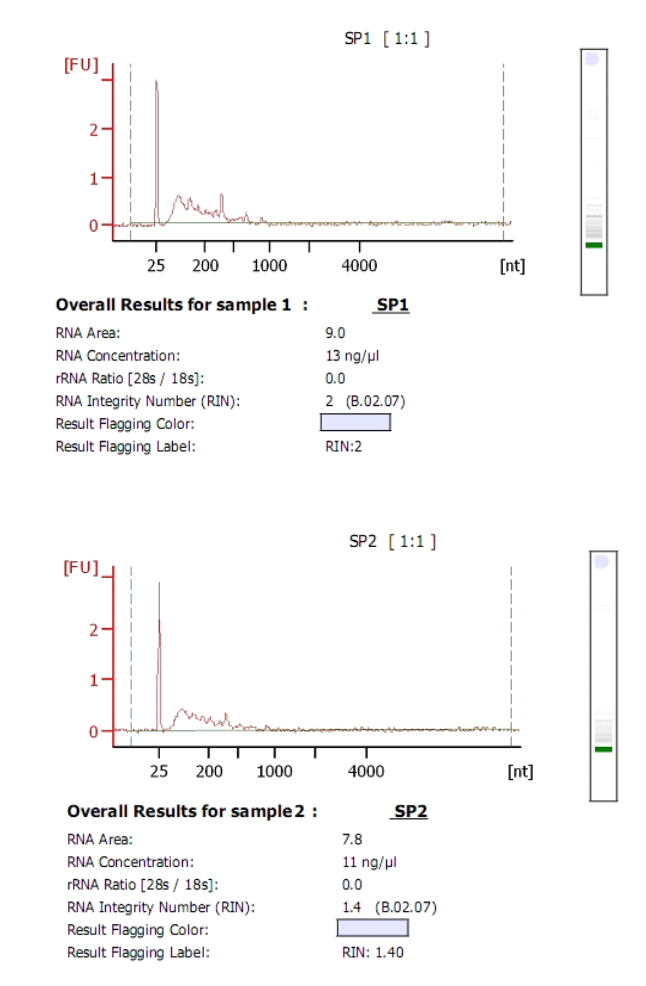

Supplement: Figure S2 — Agilent 2100 Bioanalyzer analysis of small RNA samples co-precipitated with SjAgo2 by two 27A9 IP assays. The predominant species of the small RNAs was around 25 nt. (TIF) [file pntd.0001745.s002.tif]

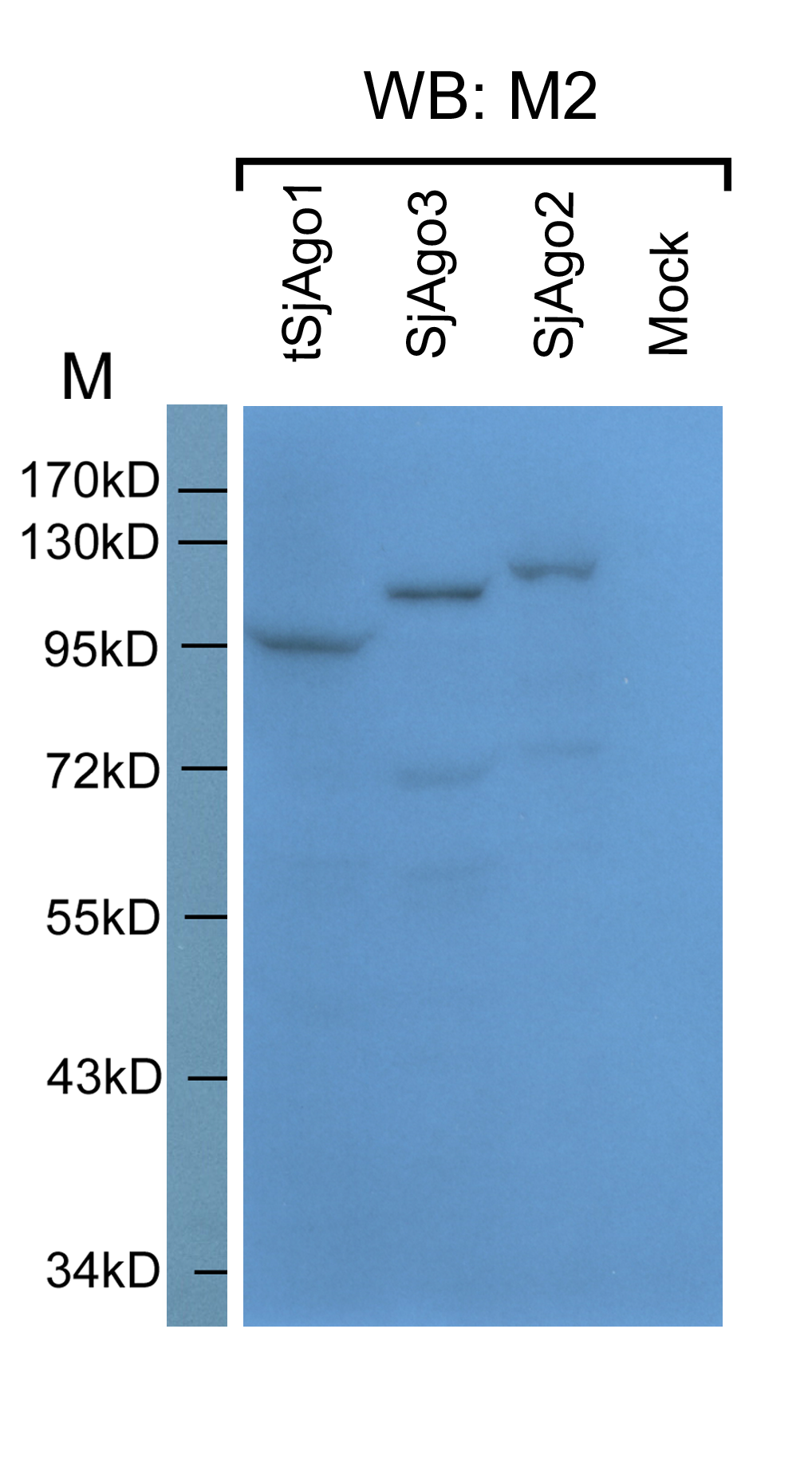

Supplement: Figure S3 — The expression of Flag-tagged SjAgos in 293T cells was detected by Western blot with mAb M2 (anti-Flag) after adjusting the loading volumes of protein samples. (TIF) [file pntd.0001745.s003.tif]

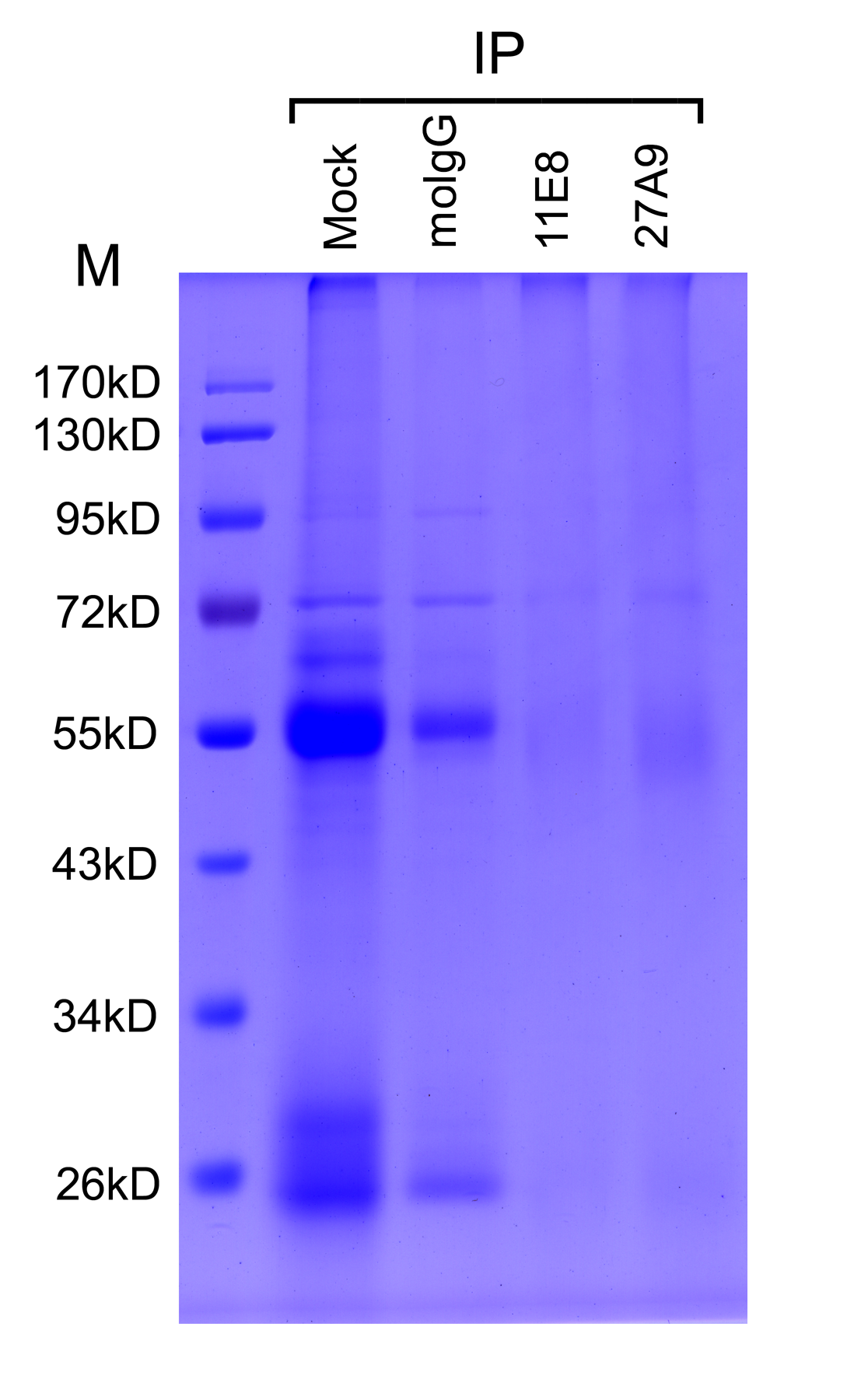

Supplement: Figure S4 — SWAP was sequentially incubated with pure Protein-A/G agarose beads only (Mock), and normal mouse IgG, mAb 11E8, and 27A9. The precipitated protein complexes were resolved on 10% SDS-PAGE, stained with Coomasie brilliant blue. (TIF) [file pntd.0001745.s004.tif]
